# Household Surveys in the General Population and Web-Based Surveys in IQOS Users Registered at the Philip Morris International IQOS User Database: Protocols on the Use of Tobacco- and Nicotine-Containing Products in Germany, Italy, and the United Kingdom (Greater London), 2018-2020

**DOI:** 10.2196/12061

**Published:** 2019-05-09

**Authors:** Zheng Sponsiello-Wang, Peter Langer, Luis Prieto, Mariia Dobrynina, Dimitra Skiada, Nathalie Camille, Rolf Weitkunat, Frank Lüdicke

**Affiliations:** 1 Philip Morris Products SA Neuchatel Switzerland

**Keywords:** cross-sectional survey, prevalence, tobacco products, tobacco heating system (THS), IQOS

## Abstract

**Background:**

Philip Morris International (PMI) has developed a novel heat-not-burn tobacco product, Tobacco Heating System (THS), which is marketed under the brand name of IQOS with HEETS (IQOS). The aerosol generated by THS has substantially fewer toxicants than combustible cigarette smoke, although the extent of the reduction of harmful and potentially harmful constituents reported varies between studies. To evaluate the potential harm reduction associated with IQOS use, the assessment of the uptake and continued use of IQOS in the context of all other tobacco- and nicotine-containing products is crucial. In March 2018, PMI launched cross-sectional surveys in Germany, Italy, and the United Kingdom (Greater London) to estimate the prevalence and use patterns of IQOS and other tobacco- and nicotine-containing products use in these 3 markets following the commercialization of IQOS. This study describes the protocol of the surveys.

**Objective:**

The objectives of these surveys are to estimate the prevalence of tobacco- and nicotine-containing products use; describe past and current patterns of use; and explore their associations with self-reported health, motivation to use, risk perceptions, and perceived aesthetic changes.

**Methods:**

The overall design of the surveys is similar in all 3 countries. Repeated cross-sectional surveys are being conducted annually over 3 consecutive years (2018 to 2020) and in 2 samples: a representative sample of the general population and a sample of IQOS users. A total of 6085 adults per year will be selected from the general population for each survey through multistage stratified sampling, and participants will respond to face-to-face computer-assisted personal interviews. In addition, 1404, 1384, and 1246 IQOS users per year in Germany, Italy, and Greater London, respectively, will be randomly selected from the PMI IQOS user database and will be invited to complete the Web-based survey using computer-assisted self-interviews. The Smoking Questionnaire is used to assess the tobacco use patterns of the participants.

**Results:**

The recruitment of the general population sample began in March 2018 and that of the IQOS user sample began in April 2018. The data collection is ongoing, and the results of the first year data analysis are expected to be available by June 2019.

**Conclusions:**

As the design of the 3 surveys is similar, the results will allow for cross-countries comparison of the prevalence of IQOS and other tobacco- and nicotine-containing products use as well as patterns of use and associated factors.

**International Registered Report Identifier (IRRID):**

DERR1-10.2196/12061

## Introduction

### Background

Tobacco smoking is the leading cause of preventable disease and is responsible for about 6 million deaths across the world each year [1]. Smokers are far more likely than nonsmokers to get heart disease, lung cancer, chronic obstructive pulmonary disease, and other diseases [2]. For smokers, the best way to reduce the harm and risks of smoking-related disease is to quit. However, nicotine is addictive, and smoking cessation has proven difficult for many smokers [2,3]. Although there is little evidence that nicotine itself causes smoking-related diseases when decoupled from smoke [4,5], in getting nicotine from smoking, smokers are exposed to enormous harm because of the inhalation of toxic components resulting from the combustion of cigarettes. When a smoker does not want to stop all nicotine use, harm minimization implies striving for the complete elimination of smoked tobacco exposure by substituting it with the use of less harmful noncombusted forms of nicotine instead of smoking [4]. Tobacco harm minimization and reduction have been increasingly recognized by the scientific society and health authorities as a valuable and promising strategy to decrease smoking-related population harm [4,6]. One of the Food and Drug Administration’s (FDA) new tobacco strategy is to recognize and clarify the role that potentially less harmful tobacco products could play in improving public health [4,5].

Toward this end, Philip Morris International (PMI) has developed a novel heat-not-burn product, Tobacco Heating System (THS). THS was designed to generate an aerosol that has substantially fewer toxicants than combustible cigarette smoke by heating the tobacco at a temperature that avoids combustion. To assess the potential health benefit of THS, a multistep assessment plan has been developed by PMI to demonstrate that (1) THS reduces harm and the risk of tobacco-related disease to individual tobacco users and (2) THS benefits the health of the population as a whole. To address the individual risk reduction associated with THS use, premarket assessment studies including aerosol chemistry, in vitro and in vivo toxicity studies, and clinical studies coherently demonstrate that switching from cigarette smoking to THS use consistently and significantly reduces the exposure to harmful and potentially harmful constituents (HPHCs) to levels that approach the reductions associated with smoking abstinence [7-15]. Numerous independent studies [16-19] and government-affiliated labs including the FDA’s Southeast Tobacco Laboratory [20], UK Committee on Toxicity [21], German Federal Institute for Risk Assessment (BfR) [22], Japan National Institute of Public Health [23], New Zeeland CRL Energy Ltd [24], and Dutch National Institute for Public Health and Environment (RIVM: Rijksinstituut voor Volksgezondheid en Milieu ) [25] have evaluated the aerosol chemistry of THS and confirmed the lower levels of HPHCs in the emissions of THS. Public Health of England [26] has systematically reviewed the existing evidence on THS and concluded that THS is likely to expose users to lower levels of HPHCs. However, higher quantities of several components other than HPHCs have been reported [27]. Auer et al [28] reported a much higher concentration of the polycyclic aromatic hydrocarbon acenaphthene for THS relative to cigarettes. According to the FDA (FDA briefing document, Meeting of the Tobacco Products Scientific Advisory Committee, 2018), the data published by Auer et al [28] are not considered adequate for comparing the levels of HPHCs between THS and combustible cigarettes. Personal communication between FDA reviewers and the paper’s authors indicated that Auer et al [28] used a smoking device designed in their facility to capture mainstream aerosol with a modified International Organization for Standardization smoking regimen to perform the analysis. In addition, the identity of some of the compounds, such as acenaphthene, cannot be confirmed as the method used is not selective. PMI premarket consumer perception and behavior evaluations on the intensity of THS use, such as abuse liability, product appeal, and consumer perception, have shown that THS is not only an acceptable alternative to cigarettes for at least part of the adult cigarette smoker population but is also properly understood by adult smokers and nonsmokers [29,30]. To address THS benefit to the health of the population as a whole, a comprehensive postmarket program including cross-sectional surveys has been established to collect relevant data on the prevalence of products use, patterns of use, and product perception. One key determinant of population harm is the assessment of the prevalence, uptake, and continued use of THS and in the context of all other tobacco- and nicotine-containing products [4,31,32]. It has been pointed out that population net exposure to harmful toxicants depends on the actual patterns and prevalence of product use [4]. The potential population health benefit of THS use would be achieved if, in addition to reduced toxicity, THS is widely accepted by smokers, does not attract nontobacco users, and does not negatively influence smokers who intend to quit.

THS, which is commercialized under the brand name of IQOS with HEETS (IQOS), was first launched in Nagoya, Japan, in 2014. Shortly after national expansion and commercialization in Japan in 2016, PMI initiated a 3-year cross-sectional survey to assess tobacco use prevalence and patterns of tobacco product use in the Japanese population [33]. The first-year survey results show that among the Japanese population, the prevalence of current use was 18.5% for any tobacco product, 17.6% for cigarettes, 1.8% for IQOS, and 0.7% for electronic cigarettes (e-cigarettes) [34]. IQOS is commercialized in more than 40 markets worldwide at present. In Italy and Germany, the product was launched in November 2014 and June 2016, respectively. In the United Kingdom, IQOS was launched in Greater London in November 2016 as a test market. To estimate the prevalence of tobacco use including IQOS and assess product use patterns in these 3 markets, PMI launched the repeated cross-sectional surveys in 2018. As in the Japanese survey, these cross-sectional surveys are being conducted in 2 samples: a representative sample of the general population and a sample of IQOS users.

### Objectives

The objectives of these surveys are to (1) estimate the prevalence of current tobacco- and nicotine-containing products use including IQOS; (2) describe product use patterns, that is, never use, initiation, product use transition, cessation, reinitiation, and relapse; (3) explore the associations between self-reported health status and use of tobacco- and nicotine-containing products in the general population as well as in a targeted sample of IQOS users in each country; and (4) among the targeted samples of IQOS users to explore the associations between patterns of tobacco- and nicotine-containing products use (including misuse) and motivation to use novel tobacco products, perceived quality attributes of IQOS (eg, risk aesthetic changes), and consumer satisfaction.

## Methods

### Survey Setting

The surveys are performed in accordance with ethical principles that have their origin in the Declaration of Helsinki [35] and are consistent with Good Epidemiological Practice [36] and International Ethical Guidelines for Epidemiological Studies [37]. Before the start of the surveys, a confirmation that approval is not required according to local laws has been obtained from the ethics committee of each market.

In all 3 markets, the cross-sectional surveys are being conducted annually in 2 samples over 3 consecutive years from 2018 to 2020: a representative sample of the general population and an IQOS user sample drawn from registered users in a PMI IQOS user database. The general population sample serves to estimate the prevalence of current tobacco- and nicotine-containing products use. The latter is chosen to be able to describe the patterns of IQOS use with an acceptable precision that is deemed to be hardly possible, with an anticipated low IQOS use prevalence in the representative general population sample shortly after product launch.

#### General Population Sample

The general population samples in the 3 markets are adults older than 18 years, living in Germany, Italy, and Greater London. The inclusion criteria are listed in Table 1. In Germany and Greater London, the subjects are randomly sampled from the general population in 3 steps: area sampling (primary sampling point), household selection, and selection of target persons (Table 1).

In Germany, census data of the Work Group of German Market and Social Research Institutes (Arbeitskreis Deutscher Markt- und Sozialforschungsinstitute e. V. ADM-Sampling-System for face-to-face surveys) are used to draw a stratified random sample. Using the ADM-Sampling system, a stratified random sample of 258 sample points is selected, taking into account the region and size of the community. From each sample point, the target households are randomly selected. Within each household, the target respondent is identified through the next birthday method, that is, the person in the household whose birthday is next.

The primary sampling points in Greater London are output areas (OA; the smallest administrative unit). A stratified random sample of 305 OAs is selected with probability proportionate to size among 32 Boroughs. From each OA, a total of 55 addresses are randomly chosen from the Postal Address File, a database that contains all known “Delivery Points” and postcodes in the United Kingdom. The target respondent in the household is selected based on the next birthday method.

In Italy, a stratified random probability sample is drawn according to a set of 3 different subpopulations or strata: (1) municipalities (140 in total), (2) electoral wards within the municipalities, and (3) individuals within the lists of the electoral wards. The sizes of the random samples from each stratum are proportional to the strata population sizes (proportionate stratification).

For each market, 6085 participants per year are to be interviewed. On the basis of initial results from the Japanese cross-sectional survey [33], assuming 1% IQOS uptake in each market, this sample size is sufficient to estimate the prevalence with a 95% confidence and a precision of ±0.25 percent points.

In Germany and Italy, the surveys are conducted as part of a face-to-face computer-assisted personal interviews (CAPI) omnibus. The omnibus is a syndicated survey with multiple participating clients, the questionnaires being divided into sections. Annually, the data are collected over 6 to 7 waves, with over 1000 participants per wave.

In Greater London, face-to-face interviews of the target participants are performed with CAPI. The annual data collection comprises 4 waves with a minimum of 1250 interviews per wave.

**Table 1:** Inclusion criteria and sample frames of the general population and IQOS users in cross-sectional surveys in Germany, Italy, and Greater London.

Samples	Sample frame	Inclusion criteria	Survey methods
General population	Area sampling (primary sampling point): 258 regions sample points in Germany, 140 municipalities in Italy, and 305 output areas in London; household (Germany and Greater London) or electoral ward selection (Italy) within the primary sampling point; and identify target persons from the household (Germany and Greater London) or from the lists of the electoral wards (Italy)	Aged ≥18 years; currently residing in Germany, Italy, or Greater London; able to read, write, and understand German, Italian, or English; and consent to participate in the survey	Face-to-face omnibus computer-assisted personal interviews (Germany and Italy) and face-to-face computer-assisted personal interviews (Greater London)
IQOS users	Random sample from PMI^a^ Germany, Italy, and UK IQOS user database	In addition to the above criteria, has used more than 100 THS^b^ tobacco sticks in his or her lifetime, is currently using IQOS, has access to the internet, and is currently not employed by PMI or any of its affiliates	Web-based; computerized assisted self-interviews

^a^PMI: Philip Morris International.

^b^THS: Tobacco Heating System.

#### IQOS User Sample

In all 3 markets, the PMI IQOS user database consists of adult smokers who purchased the IQOS devices either via a website, by telephone, or from IQOS retail stores; registered their IQOS devices in the database; and are willing to be contacted for market research purposes. IQOS users are randomly sampled from the database among those who did not participate in PMI market research panel. The participants are invited to participate in the study by email with a link to the study questionnaire. The Web-based surveys on the IQOS user sample are conducted with computerized assisted self-interviews. The inclusion criteria are listed in Table 1. As a token of appreciation, the participants receive a gift voucher of EUR 10 or GBP 10.

On the basis of initial results from the Japanese cross-sectional survey [33] that 63.4% of IQOS users fully converted to exclusive IQOS use, 1404, 1384, and 1246 IQOS users per year in Germany, Italy, and Greater London, respectively, will be surveyed. This sample size will be sufficient to estimate the proportion of fully converted IQOS users with a 95% confidence and a precision of ± 2.5 percent points.

The annual data collection of IQOS user samples is conducted in 4 waves coinciding with the waves for the general population survey.

### Survey Questionnaire

The survey questionnaire (Multimedia Appendices 1 and 2), consisting of 3 parts, was developed to address the survey objectives.

Part 1 comprises a multidimensional Smoking Questionnaire (SQ) [38,39], which was developed to standardize the assessment of cigarette smoking exposure covering the major dimensions of cigarette smoking. SQ is used to assess cigarette smoking history and behavior. Test-retest reliability of the SQ and concurrent validity with the Behavior Risk Factor Surveillance System 2011 questionnaire have demonstrated that the SQ is reliable and easy to use [39]. Most importantly, the information collected using SQ allows for classification of smoking behavior and history according to the World Health Organization (WHO) definitions [40]. It can also address dimensions of smoking behavior and history that are not covered by WHO definitions, for example, on the duration of smoking or cessation, on smoking intensity, and on smoking patterns, with regard to occasional smoking. In addition, the SQ is flexible, and it can be easily adapted to assess the exposure to other novel tobacco products such as IQOS, e-cigarettes, PLOOM, and glo. To ensure that the information related to each product is correctly understood, a description together with the pictures of each product is presented to the participants before displaying the questionnaires. Directly after the SQ for cigarette smoking, questions related to IQOS use are asked. Before assessing other novel tobacco- and nicotine-containing products, a specific question is asked to distinguish between e-cigarettes and other products such as PLOOM and glo. Information on the current use behavior regarding smokeless tobacco and other tobacco- or nicotine-containing products such as cigars, cigarillos, pipes, and water-pipes as well as nicotine-replacement therapy (NRT) products is also collected. In addition, first product use, age of initiation, and quit attempt are included in the survey questionnaire. The motivation to use IQOS and/or any other emerging tobacco products is collected according to the German Study on Tobacco Use (DEBRA: “Deutsche Befragung zum Rauchverhalten”) questionnaire [41].

The second part assesses self-reported health status, which is included to explore associations with patterns of tobacco product use. Participants are asked to rate the overall state of their health, how their health compares with the average person of their age, and how much they are worried about their health [42]. The type and extent of comorbidities are measured with a validated instrument for epidemiological studies based on self-reported outcomes [43]. In addition, the Self-Reported Changes Questionnaire, which records changes since starting using the product in a number of relevant domains where IQOS may have potential benefits (eg, teeth coloring, breath smell, exercise capacity, and skin appearance), is included for IQOS user survey.

The third part is specific to assess IQOS users’ use experience and perceived risk. The degree to which participants experience the reinforcing effects of using IQOS compared with cigarette smoking, such as satisfaction, psychological rewards, aversion, enjoyment of respiratory tract sensations, and craving reduction, is assessed with the Tobacco/Nicotine-containing Product Evaluation Questionnaire (ToNiPEQ). ToNiPEQ is an adapted version of the validated Modified Cigarette Evaluation Questionnaire [44], as proposed for smokeless tobacco [45]. Information on the type and frequency of potential misuse of THS tobacco sticks and frequency of potential misuse of the IQOS electronic device is collected with a misuse questionnaire developed by PMI. The perceived health risk of getting 18 diseases/conditions associated with smoking and using IQOS is assessed with the Perceived Risk Instrument-general version (PRI-G). PRI-G is a self-report instrument with a unidimensional scale that has been developed by PMI based on Rasch Measurement Theory and traditional psychometric methods [46].

Along with the above 3 components, the demographic characteristics of the participants including age, sex, income, education, occupation, and ethnicity are collected. In Greater London, social grade based on occupation, which is widely used for social classification in both official statistics and academic research, is rated by the interviewer for the general population survey.

In all 3 countries, the survey questionnaires are translated into local languages to minimize systematic biases because of differences in linguistic expression.

### Outcome Measures

To estimate the prevalence and describe product use patterns, the product use behavior will be categorized based on individual product use or combined product use, changes in product use behavior, and status of product use for each tobacco- and nicotine-containing product.

IQOS use patterns are classified according to the current consumption as either exclusive (no other product than IQOS), dual (IQOS and 1 more product), and poly (IQOS and ≥2 products) use, irrespective of relative consumption levels.

For the changes in product use behavior, the following behaviors will be analyzed: initiation (start using a tobacco product for the first time), relapse (start using a tobacco product again after stopping for less than 12 months), reinitiation (start using a tobacco product after having stopped for more than 12 months), and product use transition (change of consumption from 1 tobacco product to another different tobacco product), in which the transition can be further categorized based on the numbers of products use after the transition, for example, complete transition (exclusive use of switched product) or partial transition (combined use of switched product with other products).

The status of each product use will be categorized as current use, former use, and nonuse based on WHO definitions or adapted definitions. For example, for IQOS use, a current IQOS user is defined as a person who uses HEETS tobacco sticks either every day or occasionally at the time of the survey and has used 100 or more HEETS tobacco sticks in his/her lifetime. A former IQOS user is defined as a person who was formerly using IQOS either daily or occasionally but does not use IQOS at the time of the survey and has used 100 or more HEETS tobacco sticks in his/her lifetime. A non-IQOS user is a person who, at the time of the survey, does not use IQOS at all. For each status of individual product use, the use pattern will be further subcategorized according to WHO definitions. Current individual product use is subcategorized as daily or occasional use. Occasional use is subcategorized as reducer, experimenter, and continuing occasional user. Nonuse is subcategorized as ex-use, never use, and ex-occasional use. For example, a never IQOS user is a person who has never used IQOS at all or has never used IQOS daily and has used less than 100 HEETS tobacco sticks in his/her lifetime.

In addition, years between quitting cigarette smoking and initiation of IQOS, reasons for using IQOS together with subjects’ level of interest to quit, and quit attempt will be analyzed. Although the results might not be able to provide direct information on the differences between IQOS users who would not have quit smoking cigarettes and those who would have had if IQOS had not been on the market, it will allow for assessing the impact of IQOS on cigarette smoking behavior.

### Data Analysis

Response rates will be computed in accordance with the American Association for Public Opinion Research guidelines [47]. As the surveys are carried out with all households units that are contacted and are willing to participate, unit nonresponse can be distributed disproportionally and may cause biases in the sample. To account for these distortions, data will be standardized to the 2010 world population [48] and the population of each country, that is, 2011 German population, 2011 Italian population, and census data of 2011 UK population, Greater London population. Three standardizations will be performed: (1) by age, (2) by sex, and (3) by age-sex combinations. Standardized prevalence will be calculated as follows:

DSR_k_=(∑_i=1_*N*_*i*_ *P*_*ki*_*)/* (∑_i=1_*N*_*i*_*)*

1

Where DSR_k_ is the directly standardized prevalence of characteristic *k* (use of a certain tobacco product), *p*_ki_ is the observed prevalence of *k* in subgroup_i_, and N_i_ is the size of the population in subgroup_i_. The direct standardization weights the stratum-specific estimates by the size of the corresponding strata in the population.

Nonstandardized and standardized data will be analyzed and summarized descriptively. For continuous data, summary statistics will include the number and percent of subjects (n), the number of subjects with missing data, the arithmetic mean with 95% CIs, SD, median, first and third quartiles, minimum, and maximum; for log-normal data, the geometric mean and coefficient of variation will be presented. For categorical data, frequency counts, percentages, and 95% CIs will be presented. For the calculation of summary statistics and statistical analysis, unrounded data will be used.

Analyses will be stratified by the following factors: tobacco product used, sex, age, time since product initiation, intensity of use, and socioeconomic parameters (eg, income, education, and occupation). The trends in prevalence, initiation and cessation rates, as well as user behaviors will be presented based on annual data. The associations between patterns of tobacco product use and self-reported health status will be analyzed. In addition, for the population of IQOS users, the association between patterns of use (including misuse) with motivations to use, risk perceptions, perceived aesthetic changes, and consumer’s satisfaction will be explored.

## Results

In all 3 regions, the first-year data collection began in March 2018 for the general population and in April 2018 for IQOS users. We have planned to complete the first-year data collection by the end of 2018 for Germany and Italy and by the beginning of 2019 for Greater London.

Directly after the completion of the first-year data collection, the second-year data collection will start. In total, the annual data collection will be repeated for 3 consecutive years from 2018 to 2020.

The results of the first-year data analysis are expected to be available by June 2019.

## Discussion

### Strengths

The cross-sectional surveys in Germany, Italy, and Greater London, following the design of an on-going Japanese cross-sectional survey, are being conducted in representative samples of the general population and in nonrepresentative samples of registered IQOS users. Additional improvements have been implemented, including (1) sampling methods, (2) sample size calculation, and (3) survey questionnaire. For the general population sample, a random probability population-based sampling approach is employed instead of nonprobabilistic quota sampling as in the Japanese cross-sectional survey. Thus, the samples are more likely to be representative of the population. The sample size calculation of the surveys in 3 markets takes into account the results of the Japanese survey, that is, the IQOS use prevalence is about 1% IQOS in the general population and 63.4% IQOS users use IQOS exclusively in the IQOS sample. This sample size is sufficiently large to allow for the accurate estimates of tobacco use prevalence at the national level. Furthermore, we have implemented a new survey questionnaire. The SQ included in the surveys allows for a comprehensive characterization of tobacco use histories and behaviors according to WHO definitions [40]. By adapting the SQ, the exposure to other novel tobacco products available on the local markets such as IQOS, e-cigarettes, PLOOM, and glo can be assessed. However, measuring the use of novel tobacco products is challenging. As these products have not been on the market long enough, there is no established history of these novel products in surveillance surveys, and often, product-specific nomenclature and measures are not standardized. Most of the ongoing population-based smoking surveys either do not include these products yet and/or do not include exhaustive lists of tobacco products available to the public. In the these surveys, the participants have the opportunity to report all novel tobacco- and nicotine-containing products used, allowing a comprehensive assessment of the exposure to any type of tobacco- and nicotine-containing products. Although the accuracy of the SQ for cigarette smoking has been assessed, the adapted version for other novel tobacco products has not been validated. For the novel products that use tobacco sticks, such as IQOS and glo, the use behavior is quantified in a similar way as cigarette smoking. However, assessing exposure of vaporing products, either with electronic liquid such as e-cigarette or tobacco capsule such as PLOOM tech, is challenging. Vaporing behavior is different from cigarette smoking, as it is not bounded by the time it takes to burn a cigarette. Puffing number, frequency, and duration vary among the users. For e-cigarettes, different device designs and numerous varieties in nicotine levels and compositions are making the use pattern comparisons with other tobacco products even more difficult. Currently, there is no standard tool for assessing vaporing products exposure, although core items for assessing e-cigarette use in population-based surveys have been recommended by Pearson et al [49]. Our survey results on the use behavior of different products will provide valuable information regarding the feasibility of the adapted SQ.

In addition to measuring tobacco use patterns, these surveys will also collect information on motivation to use IQOS and intent to quit, which will allow for establishing the impact of IQOS on cigarette smoking behavior. The surveys will provide data on the attractiveness and acceptability of IQOS in current IQOS users as well as the potential effects of IQOS use on the intention of adult smokers to quit smoking. To assess the role of IQOS in harm reduction, evidence is needed to determine if IQOS has the potential to successfully compete with and replace smoking. Furthermore, these surveys will characterize the profile of dual IQOS users. According to a large, nationally representative, longitudinal study of tobacco use and health in the United States [50], more than a quarter of adults are current users of at least one type of tobacco product. There have been concerns that dual use, for example, dual use of cigarette and e-cigarette, may undermine cigarette smoking cessation, or worse, increase smoking and nicotine dependence [51,52]. However, more and more evidence indicates that e-cigarette use may promote smoking cessation and reduction among dual users [53-56], and dual use of tobacco and e-cigarettes does not necessarily perpetuate or exacerbate smokers’ tobacco addiction and use [54]. Currently, we do not know if IQOS use is associated with quitting or reducing smoking in current smokers or dual IQOS users and contributing to relapse prevention in former smokers. If IQOS use has a similar effect as e-cigarettes on smoking cessation and relapse [26,53,57-63], the potential role of IQOS on smoking cessation intervention might be inferred.

In the surveys, the perceived health risk associated with IQOS use will be compared with that of smoking. Risk perceptions related to emerging products possibly have an important influence on how emerging nicotine products are used and by whom [64]. Currently, there are misperceptions of emerging nicotine products, such as e-cigarettes, regarding the relative harmfulness. It has been shown that in the United Kingdom, only half of the adult smokers believe that e-cigarettes are less harmful than smoking and a majority of smokers and ex-smokers do not think that completely switching from cigarettes to e-cigarettes would lead to major health benefits [26]. The misperception is most likely linked to the perception that most adverse health effects are caused by nicotine [26]. It has been shown that half of the smokers even overestimate the harmfulness of NRTs [26]. Lack of knowledge about the products’ health effects may contribute to the misperceptions [64]. Other studies have shown that misperceptions vary consistently by indicators of socioeconomic status, with more disadvantaged smokers and recent ex-smokers having higher rates of misperceptions [26]. The consequence of overestimating the harmfulness of NRTs and e-cigarettes is that smokers may be discouraged from using them in an effort to quit smoking [64]. Public Health England has pointed out that future research should aim at assessing the causes and effects of misperceptions of the relative harmfulness of e-cigarettes and NRTs compared with cigarettes [26]. Compared with e-cigarettes, which contain mainly nicotine liquids, the aerosol generated from THS contains not only nicotine but also other HPHCs, although significantly reduced, compared with cigarette smoke. Consequently, it is likely that the perceived harmfulness of THS will be overestimated, which could impact the acceptance and usage of the product. The survey results will provide evidence on risk perceptions of IQOS users under real-world conditions, although our premarketing assessment has demonstrated that the perceived risk associated with THS is lower than that associated with cigarettes, which is the most hazardous tobacco product, and higher than that associated with NRTs or cessation. The higher risk perception of THS compared with NRTs [29] is consistent with existing literature on risk perception of novel products compared with NRTs [65,66].

Although the weight of evidence that IQOS significantly reduces the exposure to HPHCs is compelling [7-15], higher levels of several components other than HPHCs are found in the aerosol generated by THS. Currently, there is no direct evidence available on the risk reduction of IQOS, and the health impact of the components other than HPHCs is unknown. Recently, Public Health England conducted a systematic review of available evidence and concluded that heated tobacco products may be considerably less harmful than tobacco cigarettes [26]. Our survey results on the perceived general health status [42], self-reported comorbidity [43], and, in particular, the self-reported changes in a number of relevant domains where IQOS users may have potential benefits since starting using the product will allow for exploring the potential health benefits of IQOS.

### Limitations

These surveys have some limitations. The cross-sectional studies will not collect prospective data on product-use transition such as changes in the frequency and intensity. However, the SQ, which captures tobacco use history from past 3 months to more than 20 years ago, can provide useful retrospective information on tobacco use. Caution should be taken when evaluating the data, as recall bias and self-reporting bias could impact the data quality and limit the conclusiveness of the results. As IQOS was launched very recently in these 3 markets, a low IQOS use prevalence in the general population sample is expected. The sample size calculation is based on the assumption of 1% IQOS uptake. In case of even lower IQOS use prevalence, the studies are underpowered. However, reasonable inferences can still be made by incorporating information from CIs. The surveys are not designed to claim any causal effects of using tobacco- and nicotine-containing products on self-reported health. The assessment of self-reported, perceived aesthetic changes and current health status will rather allow to characterize different user populations accordingly. Nevertheless, the results of the surveys will provide useful information on the associations between patterns of tobacco product use and associated factors. Furthermore, the limitation of the IQOS user database is that it is most likely not representative of the IQOS user population in each country, as not all IQOS users who purchased the devices are registered in the database. There is also a chance of unintended over-representing of IQOS enthusiasts in the consumer database. Thus, the findings from the IQOS samples cannot be generalized to populations outside the samples, and across-country comparisons are limited. The comparison of IQOS user profiles sampled from the database with those sampled from the general population will provide valuable information regarding the extent of the nonrepresentativeness. However, this comparison is possible only when the IQOS use prevalence in each market has reached a sufficient level. In addition, the number of potential IQOS participants in the IQOS sample is highly dependent on the size of the PMI IQOS database. This might be critical for the Greater London survey, in particular for the first few waves, as the IQOS database in Greater London is currently relatively small compared with those in Italy and Germany. The risk of not being able to enroll a sufficient number of IQOS participants during the first few waves cannot be fully excluded.

### Conclusions

The surveys aim to assess the prevalence of tobacco use and will provide insights into use patterns and associated factors. As the surveys will be conducted in 3 markets with similar design and at regular intervals, the results will allow for cross-regional and trend assessments. Most importantly, the results will provide relevant information allowing for assessing the potential health benefit of IQOS in the population.

## Appendix

Multimedia Appendix 1 UK general population sample survey questionnaire.

Multimedia Appendix 2 UK IQOS user sample survey questionnaire.

## Abbreviations

CAPI: computer-assisted personal interviews
e-cigarette: electronic cigarette
FDA: Food and Drug Administration
HPHCs: harmful and potentially harmful constituents
NRT: nicotine-replacement therapy
OA: output area
PMI: Philip Morris International
PRI-G: Perceived Risk Instrument-general version
SQ: Smoking Questionnaire
THS: Tobacco Heating System
ToNiPEQ: Tobacco/Nicotine-containing Product Evaluation Questionnaire
WHO: World Health Organization
