# Supplementary material for: Household Surveys in the General Population and Web-Based Surveys in IQOS Users Registered at the Philip Morris International IQOS User Database: Protocols on the Use of Tobacco- and Nicotine-Containing Products in Germany, Italy, and the United Kingdom (Greater London), 2018-2020
Source: JMIR Res Protoc. 2019 May 9;8(5):e12061. doi: 10.2196/12061 (PMC6532333; doi:10.2196/12061)
Supplement: Multimedia Appendix 1 [file resprot_v8i5e12061_app1.docx]

**Appendix 1 Cross-Sectional Survey Questionnaire for the General Population in Greater London**

**SCREENING & DEMOGRAPHIC QUESTIONS**

**[ASK ALL, SP]**

**UK_S1: Which of the following describes how you think of yourself?**

**PLEASE SELECT ONE ANSWER**

1. Female
2. Male
3. In some other way

**[ASK ALL, RANGE 1-110]**

**UK_S2: What is your age in years?**

**PLEASE TYPE IN BELOW**

└─┴─┘years

**[close & thank if under 18 years oF age]**

**[ASK ALL, SP]**

**UK_S3: And is this your main residence?**

**PLEASE SELECT ONE ANSWER**

1. Yes
2. No

**[close & thank if NO]**

**[DO NOT ASK, AUTOCODE AS YES IF UK_S3=1]**

**UK_S3a: Do you live in the Greater London area?**

1. Yes
2. No

**[close & thank if not living in greater london]**

**[DO NOT ASK, AUTOCODE BOROUGH FROM SAMPLE POINT IF UK_S3=1]**

**UK_S3b: In which London Borough do you live?**

1. Barking and Dagenham
2. Barnet
3. Bexley
4. Brent
5. Bromley
6. Camden
7. Croydon
8. Royal Borough of Greenwich
9. Hackney
10. Hammersmith and Fulham
11. Islington
12. Ealing
13. Enfield
14. Haringey
15. Harrow
16. Havering
17. Hillingdon
18. Hounslow
19. Royal Borough of Kensington and Chelsea
20. Royal Borough of Kingston upon Thames
21. Lambeth
22. Lewisham
23. Merton
24. Newham
25. Redbridge
26. Richmond upon Thames
27. Southwark
28. Sutton
29. Tower Hamlets
30. Waltham Forest
31. Wandsworth
32. Westminster
33. none

**[close & thank if NONE]**

**[CLOSING & THANK MESSAGE]**

**“Thank you for your interest in this study. However, based on the information provided, you do not qualify for participation. Thank you for your cooperation up to this point”**

**[DISPLAY INFORMATION SCREEN]**

**Tobacco Use Questionnaire**

**[SECTION HEADING ABOVE NOT TO BE DISPLAYED TO PARTICIPANT]**

**[DISPLAY INSTRUCTION AND INFORMATION SCREEN TO ALL PARTICIPANTS]**

| **Manufactured or roll-your-own cigarettes** | 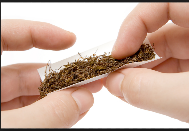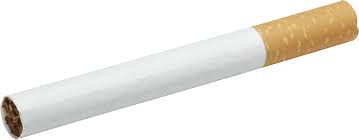 | These products contain tobacco, are lit up and produce smoke to inhale. |
| --- | --- | --- |
| **IQOS with HEETS** | 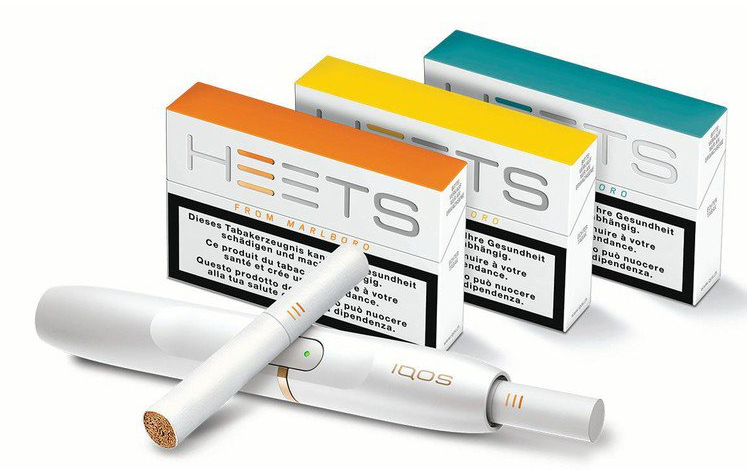 | HEETS tobacco sticks are placed in the IQOS electronic device that heats the tobacco to produce an aerosol. This is not an electronic cigarette. |
| **Other Innovative products**  *(such as electronic cigarettes*  *or similar products)* | 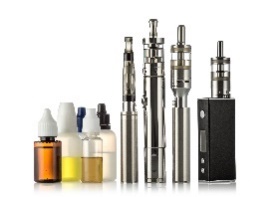 | These products can be battery-operated and contain either nicotine or tobacco. |
| **Smokeless tobacco**  *(such as chewing tobacco, snus, snuff, dissolvable)* | 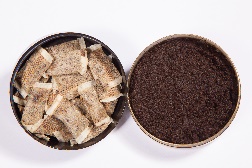 | These products are made of processed tobacco. They can be in pouches or loose. The tobacco is placed directly into the mouth or nose. |
| **Other tobacco or nicotine-containing products**  *(such as cigars, cigarillos, pipes, water-pipes)* | 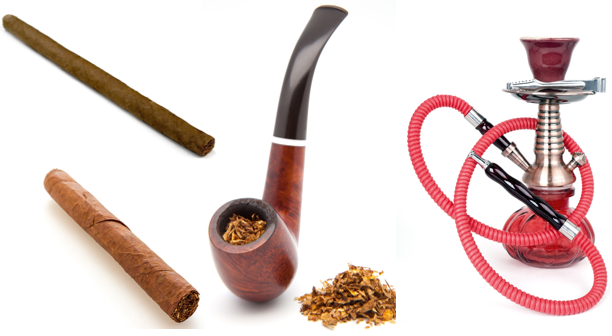 | These products come in different forms and shapes and they all contain tobacco, are lit up and produce smoke to inhale. |
| **Nicotine Replacement Therapy products also known as NRTs**  *(such as, patch, gum, tablet, inhaler, lozenge, pill)* | 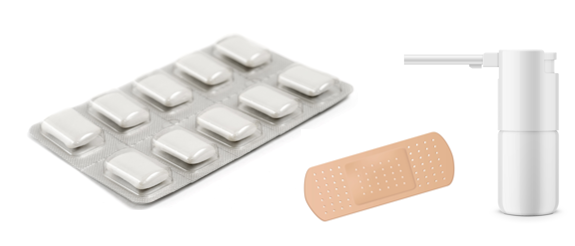 | These products deliver nicotine in controlled amounts to help people quit smoking. They are used for a limited time to help people become used to not smoking. |

**Below is some information about different products that contain tobacco or nicotine. Please review and read the descriptions below before continuing with the questionnaire.**

**CIGARETTES**

**[SECTION HEADING ABOVE NOT TO BE DISPLAYED TO PARTICIPANT]**

**The next few questions are about any experiences with smoking cigarettes. [SHOW PRODUCT 1 IMAGE AND TEXT]**

**[ASK ALL, SP]**

**QC1: What is your current cigarette smoking behaviour (including hand-rolled cigarettes)?**

**PLEASE SELECT ONE ANSWER**

1. Daily smoker (at least one cigarette per day, disregarding religious fasting)
2. Occasional smoker (less than one cigarette per day)
3. Ex-smoker of cigarettes
4. Non-smoker of cigarettes

**[ASK OCCASIONAL SMOKER ONLY (QC1=2), NUMERIC, RANGE 0-30]**

**QC1b: During the past 30 days, on how many days, if any, did you smoke?**

**PLEASE TYPE IN BELOW**

__days

**[DISPLAY IF NON-SMOKER (QC1=4)]**

**You indicated that you are a non-smoker, but just to check …**

**[ASK ALL, SP]**

**QC2: Have you ever smoked 100 cigarettes or more in your life?**

**PLEASE SELECT ONE ANSWER**

1. Yes
2. No

**[DISPLAY IF CURRENT DAILY SMOKER (QC1=1)]**

**You indicated that you are a daily smoker, but just to check, do you, or …**

**[DISPLAY IF NON-SMOKER (QC1=4)]**

**And just to check …**

**[ASK ALL, SP]**

**QC3: Did you ever smoke cigarettes regularly, i.e. at least 1 cigarette per day?**

**PLEASE SELECT ONE ANSWER**

1. Yes
2. No

**[ASK IF EVER SMOKED CIGARETTES REGULARLY (QC1=1 OR QC3=1), NUMERIC, RANGE 1 - CURRENT AGE]**

**QC4: At what age did you start to smoke regularly, i.e. at least 1 cigarette per day?**

**PLEASE TYPE IN BELOW**

└─┴─┘years **[IF <= 15 SHOW POP UP MESSAGE: “Please confirm the age you have entered is correct and click next to continue" and allow to confirm this and proceed]**

Don’t know / can’t remember

**[ASK IF EX-SMOKER (QC1=3), NUMERICS, YEAR RANGE 0 – CURRENT AGE, MONTH RANGE 0-11, DAY RANGE 0-30]**

**QC5: For how long have you quit now?**

**PLEASE PROVIDE AS MUCH INFORMATION AS POSSIBLE BY ENTERING THE YEARS, MONTHS AND DAYS AS APPROPRIATE**

└─┴─┘years plus └─┴─┘months plus └─┴─┘days

Don’t know / can’t remember

**[DISPLAY IF CURRENT OR OCCASIONAL SMOKER EVER SMOKING CIGARETTES REGULARLY (QC1=1 OR (QC1=2 AND QC3=1))]**

**Please now think about any periods when you may have quit regular cigarette smoking …**

**[DISPLAY IF EX-SMOKER OR NON-SMOKERS THAT HAVE SMOKED 100 CIGARETTES OR MORE THAT REGULARLY SMOKED ((QC1=3 OR (QC1=4 AND QC2=1)) AND QC3=1)]**

**Please now think about the time you first began smoking to the present day …**

**[ASK IF EVER SMOKED CIGARETTES REGULARLY (QC1=1 OR QC3=1), NUMERICS, YEAR RANGE 0 – CURRENT AGE, MONTH RANGE 0-11, DAY RANGE 0-30]**

**QC6: [IF CURRENT SMOKER EVER SMOKING CIGARETTES REGULARLY (QC1=1 OR (QC1=2 AND QC3=1))”If you ever quit regular cigarette smoking: “]For how long did you quit altogether? Please add together any separate periods of quitting [IF EX-SMOKER THAT REGULARLY SMOKED (QC1=3 AND QC3=1)”, including the current period since you stopped smoking”].**

**PLEASE PROVIDE AS MUCH INFORMATION AS POSSIBLE BY ENTERING THE YEARS, MONTHS AND DAYS AS APPROPRIATE [IF CURRENT OR OCCASIONAL SMOKER EVER SMOKING CIGARETTES REGULARLY (QC1=1 OR (QC1=2 AND QC3=1)) “. IF YOU HAVE NEVER QUIT PLEASE ENTER ZERO BELOW”]**

└─┴─┘years plus └─┴─┘months plus └─┴─┘days

Don’t know / can’t remember

**[DISPLAY IF EX-SMOKER OR NON-SMOKER THAT HAS SMOKED 100 CIGARETTES OR MORE (QC1=3 OR (QC1=4 AND QC2=1))]**

**Thinking now about the time when you smoked …**

**[ASK ALL DAILY, OCCASIONAL OR EX-SMOKERS, OR NON-SMOKERS THAT HAVE SMOKED 100 CIGARETTES OR MORE (QC1=1-3 OR (QC1=4 AND QC2=1)), TEXT]**

**QC7: What brand of cigarettes/hand-rolled tobacco did you predominantly smoke in the last 12 months of smoking? PLEASE TYPE IN BRAND**

Name of brand:____

Don’t know / can’t remember

**[ASK ALL DAILY, OCCASIONAL OR EX-SMOKERS, OR NON-SMOKERS THAT HAVE SMOKED 100 CIGARETTES OR MORE (QC1=1-3 OR (QC1=4 AND QC2=1))]**

**QC8: On average, how many cigarettes do/did you smoke per day?**

**Please indicate if you were on average smoking none, less than 1 per day, or the number of cigarettes per day at each of the time points below**

**[COLUMNS]**

1. none
2. less than 1 per day
3. └─┴─┴─┘per day **[RANGE 1-120]**

**[ROWS, ONE ANWER PER ROW]**

**[SUPRRESS ANY ROWS WHERE PARTICIPANT WOULD HAVE BEEN AGED 0 OR BELOW]**

1. Currently (last 3 months)
2. 1 year ago
3. 2 years ago
4. 5 years ago
5. 10 years ago
6. 15 years ago
7. 20 years ago
8. More than 20 years ago

**[ASK DAILY AND OCCASIONAL SMOKERS (QC1=1-2), SP]**

**QC9: In total, how many times, if any, in the past 12 months have you tried to quit smoking completely?**

**PLEASE SELECT ONE ANSWER**

1. I did not try to quit in the past 12 months
2. Please type in number of time(s)___ **[RANGE 1-365]**
3. Don’t know / can’t remember

**[ASK ALL WHO TRIED TO QUIT SMOKING COMPLETELY AT LEAST ONCE (QC9=2), NUMERICS, MONTH RANGE 0-11, DAY RANGE 0-30]**

**QC10: The last time you stopped smoking in the past 12 months because you were trying to quit completely, how long did you stop in total?**

**PLEASE PROVIDE AS MUCH INFORMATION AS POSSIBLE BY ENTERING MONTHS AND DAYS AS APPROPRIATE**

└─┴─┘months plus └─┴─┘days

Don’t know / can’t remember

**[ASK DAILY AND OCCASIONAL SMOKERS (QC1=1-2), SP]**

**QC11: Are you considering quitting smoking during the next 6 months?**

**PLEASE SELECT ONE ANSWER**

1. Yes, plan to stop within next 30 days
2. Yes, plan to stop within next 6 months, but not within next 30 days
3. No, not thinking of quitting in next 6 months
4. Don’t know / couldn’t say

**[ASK ALL EX-SMOKERS OF CIGARETTES (QC1=3), MP 1-5, SP 6]**

**QC12: Which of the following products did you use to help you quit smoking, if any?**

**PLEASE SELECT ALL THAT APPLY**

**[show product 2-6 imageS alongside text]**

1. IQOS with HEETS
2. Other Innovative products (such as electronic cigarettes or similar products)
3. Smokeless Tobacco (such as chewing tobacco, snus, snuff, dissolvable)
4. Other tobacco or nicotine-containing products (such as cigars, cigarillos, pipes, water-pipes)
5. Nicotine Replacement Therapy products also known as NRTs (such as, patches, gums, tablets, inhalers, lozenges, pills)
6. None

**IQOS with HEETS**

**[SECTION HEADING ABOVE NOT TO BE DISPLAYED TO PARTICIPANT]**

**The next few questions are about any experiences using IQOS with HEETS, which became available in this country in late 2016. (show product 2 image and text)**

**[ASK ALL, SP]**

**QI1: What is your current IQOS with HEETS use behaviour?**

**PLEASE SELECT ONE ANSWER**

1. Daily use (at least one HEETS per day, disregarding religious fasting)
2. Occasional use (less than one HEETS per day)
3. Ex-user of IQOS with HEETS
4. Non-user of IQOS with HEETS

**[ASK OCCASIONAL IQOS USER ONLY (QI1=2), NUMERIC, RANGE 0-30]**

**QI1b: During the past 30 days, on how many days, if any, did you use IQOS with HEETS?**

**PLEASE TYPE IN BELOW**

__days

**[DISPLAY IF NON-USER (QI1=4)]**

**You indicated that you are a non-user of IQOS with HEETS, but just to check …**

**[ASK ALL, SP]**

**QI2: Have you ever used 100 HEETS or more in your life?**

**PLEASE SELECT ONE ANSWER**

1. Yes
2. No

**[DISPLAY IF CURRENT USER (QI1=1)]**

**You indicated that you are a daily user of IQOS with HEETS, but just to check, do you, or …**

**[DISPLAY IF NON-USER (QI1=4)]**

**And just to check …**

**[ASK ALL, SP]**

**QI3: Did you ever use IQOS with HEETS regularly, i.e. at least 1 HEETS stick per day?**

**PLEASE SELECT ONE ANSWER**

1. Yes
2. No

**[DISPLAY IF EVER USED IQOS REGULARLY (QI1=1 OR QI3=1)]**

**As previously mentioned, IQOS with HEETS became available in this country in late 2016.**

**[ASK IF EVER USED IQOS REGULARLY (QI1=1 OR QI3=1), NUMERIC, RANGE 1 - CURRENT AGE]**

**QI4: At what age did you start to use IQOS with HEETS regularly, i.e. at least 1 HEETS stick per day?**

**PLEASE TYPE IN BELOW**

└─┴─┘years **[IF <= 15 SHOW POP UP MESSAGE: “Please confirm the age you have entered is correct and click next to continue" and allow to confirm this and proceed]**

Don’t know / can’t remember

**[DISPLAY EX-USER NEVER USING REGULARLY (QI1=3 AND QI3=2)]**

**As previously mentioned, IQOS with HEETS became available in this country in late 2016.**

**[ASK IF EX-USER (QI1=3), NUMERICS, YEAR RANGE 0 – CURRENT AGE, MONTH RANGE 0-11, DAY RANGE 0-30]**

**QI5: For how long have you stopped your use of IQOS with HEETS now?**

**PLEASE PROVIDE AS MUCH INFORMATION AS POSSIBLE BY ENTERING THE YEARS, MONTHS AND DAYS AS APPROPRIATE**

└─┴─┘years plus └─┴─┘months plus └─┴─┘days

Don’t know / can’t remember

**[DISPLAY IF CURRENT OR OCCASIONAL USER EVER USING IQOS WITH HEETS REGULARLY (QI1=1 OR (QI1=2 AND QI3=1))]**

**As previously mentioned, IQOS with HEETS became available in this country in late 2016. Please now think about any periods when you may have stopped regularly using IQOS with HEETS …**

**[DISPLAY IF EX-USER OR NON-USER THAT HAVE USED 100 HEETS OR MORE THAT REGULARLY USED IQOS WITH HEETS ((QI1=3 OR (QI1=4 AND QI2=1)) AND QI3=1)]**

**As previously mentioned, IQOS with HEETS became available in this country in late 2016. Please now think about the time you first began using IQOS with HEETS to the present day …**

**[ASK IF EVER USED IQOS REGULARLY (QI1=1 OR QI3=1), NUMERICS, YEAR RANGE 0 – CURRENT AGE, MONTH RANGE 0-11, DAY RANGE 0-30]**

**QI6: [IF CURRENT USER EVER USING IQOS WITH HEETS REGULARLY (QI1=1 OR (QI1=2 AND QI3=1))” If you ever stopped regular use of IQOS with HEETS: “]For how long did you stop altogether? Please add together any separate periods of stopping [IF EX-USER THAT REGULARLY USED (QI1=3 AND QI3=1)”, including the current period since you stopped your use of IQOS with HEETS”].**

**PLEASE PROVIDE AS MUCH INFORMATION AS POSSIBLE BY ENTERING THE YEARS, MONTHS AND DAYS AS APPROPRIATE [IF CURRENT OR OCCASIONAL USER EVER USING IQOS WITH HEETS REGULARLY (QI1=1 OR (QI1=2 AND QI3=1)) “. IF YOU HAVE NEVER STOPPED PLEASE ENTER ZERO BELOW”]**

└─┴─┘years plus └─┴─┘months plus └─┴─┘days

Don’t know / can’t remember

**[DISPLAY IF EX-USER OR NON-USER THAT HAS USED 100 HEETS OR MORE (QI1=3 OR (QI1=4 AND QI2=1))]**

**Thinking now about the time when you used IQOS with HEETS …**

**[ASK ALL DAILY, OCCASIONAL OR EX-USERS, OR NON-USERS THAT HAVE USED 100 HEETS OR MORE (QI1=1-3 OR (QI1=4 AND QI2=1)), SP]**

**QI7: What HEETS flavour did you predominantly use in the last 12 months of use?**

**PLEASE SELECT ONE ANSWER**

1. Regular / no flavour
2. Menthol
3. Other flavour
4. Don’t know / can’t remember

**[ASK ALL DAILY, OCCASIONAL OR EX-USERS, OR NON-USERS THAT HAVE USED 100 HEETS OR MORE (QI1=1-3 OR (QI1=4 AND QI2=1))]**

**As previously mentioned, IQOS with HEETS became available in this country in late 2016.**

**QI8: On average, how many HEETS do/did you use per day?**

**Please indicate if you were on average using none, less than 1 per day, or the number of heets per day at each of the time points below**

**[COLUMNS]**

1. none
2. less than 1 per day
3. └─┴─┴─┘per day **[RANGE 1-200]**

**[ROWS, ONE ANSWER PER ROW]**

**[SUPRRESS ANY ROWS WHERE PARTICIPANT WOULD HAVE BEEN AGED 0 OR BELOW]**

1. Currently (last 3 months)
2. 1 year ago
3. 2 years ago
4. 5 years ago
5. 10 years ago
6. More than 10 years ago

**[ASK DAILY AND OCCASIONAL USERS (QI1=1-2), SP]**

**QI9: In total, how many times, if any, in the past 12 months have you tried to stop your use of IQOS with HEETS completely?**

**PLEASE SELECT ONE ANSWER**

1. I did not try to stop in the past 12 months
2. Please type in number of time(s)___ **[RANGE 1-365]**
3. Don’t know / can’t remember

**[ASK ALL WHO TRIED TO STOP USING IQOS WITH HEETS AT LEAST ONCE (QI9=2), NUMERICS, MONTH RANGE 0-11, DAY RANGE 0-30]**

**QI10: The last time you stopped your use of IQOS with HEETS in the past 12 months because you were trying to stop completely, how long did you stop in total?**

**PLEASE PROVIDE AS MUCH INFORMATION AS POSSIBLE BY ENTERING MONTHS AND DAYS AS APPROPRIATE**

1. └─┴─┘months plus └─┴─┘days
2. Don’t know / can’t remember

**[ASK CURRENT DAILY AND OCCASIONAL USERS (QI1=1-2), SP]**

**QI11: Are you considering stopping use of IQOS with HEETS in the next 6 months?**

**PLEASE SELECT ONE ANSWER**

1. Yes, plan to stop within next 30 days
2. Yes, plan to stop within next 6 months, but not within next 30 days
3. No, not thinking of stopping in next 6 months
4. Don’t know / couldn’t say

**[ASK DAILY AND OCCASIONAL USERS (QI1=1-2), MP 1-17, SP 18]**

**QI12: We are now interested in the reason(s) why you are using IQOS with HEETS.**

**PLEASE REVIEW THE LIST OF POSSIBLE REASONS BELOW AND SELECT ALL THAT APPLY**

**I use IQOS with HEETS...**

**[RANDOMISE CODES 1-16]**

- 1. To quit tobacco use completely
  2. To quit cigarette smoking
  3. To reduce cigarette smoking without quitting completely
  4. Because I believe it is less harmful than smoking cigarettes
  5. Because I believe it is less addictive than smoking cigarettes
  6. Because I believe it reduces the urge to smoke
  7. Because I use it at locations where smoking cigarettes is prohibited
  8. Out of curiosity
  9. Because it is fun
  10. Because of the various flavours available
  11. Because friends, family or colleagues do so too
  12. Because it tastes better to me than smoking cigarettes
  13. Because it is cool/modern
  14. Because I believe it bothers other people less than smoking cigarettes
  15. Because it is hard for me to stop using IQOS
  16. Because people in the media or famous personalities use IQOS
  17. Other reasons
  18. Don’t know / couldn’t say

**OTHER INNOVATIVE PRODUCTS**

**[SECTION HEADING ABOVE NOT TO BE DISPLAYED TO PARTICIPANT]**

**[DISPLAY TEXT IF ANY EXPERIENCES OF USING IQOS (QI1=1-3 OR QI2=1 OR QI3=1)]**

**You said that you had some experience of using IQOS with HEETS. We are now interested in any other products you may have used other than IQOS.**

**[DISPLAY TEXT TO ALL]**

**The next few questions are about any experiences of using other innovative products such as electronic cigarettes, vaping devices or similar products. [SHOW PRODUCT 3 IMAGE AND TEXT]**

**[DISPLAY TEXT IF ANY EXPERIENCES OF USING IQOS (QI1=1-3 OR QI2=1 OR QI3=1)]**

**Not including IQOS …**

**[ASK ALL, SP]**

**QO1a: Have you ever used electronic cigarettes, vaping devices or other innovative products e.g. Ploom PAX etc.?**

**PLEASE SELECT ONE ANSWER**

1. Yes
2. No

**[ASK ALL USING E-CIGARETTES/INNOVATIVE PRODUCTS (QO1a=1), MP]**

**QO1b: Which such product(s) have you ever used?**

**PLEASE SELECT ALL THAT APPLY**

1. Electronic cigarettes or vaping devices
2. Other innovative products (TYPE IN NAME OR TYPE OF PRODUCT): ____________

**[ASK ALL FOLLOWING QUESTIONS FOR EACH PRODUCT TYPE AT QO1b. IF BOTH ELECTRONIC CIGARETTES AND OTHER INNOVATIVE PRODUCTS USED ASKED ALL QUESTIONS IN THE SECTION FOR ELECTRONIC CIGARETTES BEFORE LOOPING THROUGH THE SECTION AGAIN FOR OTHER INNOVATIVE PRODUCT].**

**[IF QO1b=1 DISPLAY] The next few questions are about your experiences of using electronic cigarettes. [xxx =“electronic cigarettes or vaping devices” TO BE INSERTED IN QUESTIONS BELOW]**

**[IF QO1b=2 DISPLAY] You indicated that you have used other innovative products and said this was xxx [INSERT VERBATIM ENTERED AT QO1b_2]. The next few questions are about your experiences of using this.**

**[xxx =“VERBATIM ENTERED AT QO1b_2” TO BE INSERTED IN QUESTIONS BELOW]**

**[ASK USING ELECTRONIC CIGARETTES OR OTHER INNOVATIVE PRODUCTS (QO1b=1-2), SP]**

**QO2: What is your current [xxx] use behaviour?**

**PLEASE SELECT ONE ANSWER**

1. Daily use (using **[**xxx**]** at least once per day, disregarding religious fasting)
2. Occasional use (using **[**xxx**]** less than once per day)
3. Ex-user of **[**xxx**]**
4. Non-user of **[**xxx**]**

**[ASK OCCASIONAL USER ONLY (QO2=2), NUMERIC, RANGE 0-30]**

**QO2b: During the past 30 days, on how many days, if any, did you use [xxx]?**

**PLEASE TYPE IN BELOW**

__days

**[DISPLAY IF NON-USER (QO2=4)]**

**You indicated that you are a non-user, but just to check …**

**[ASK USERS OF INNOVATIVE PRODUCTS (QO1b=1-2), SP]**

**QO3: Have you ever used [xxx] 100 times or more in your life?**

**PLEASE SELECT ONE ANSWER**

1. Yes
2. No

**[DISPLAY IF CURRENT USER (QO2=1)]**

**You indicated that you are a daily user of [xxx], but just to check, do you, or …**

**[DISPLAY IF NON-USER (QO2=4)]**

**And just to check …**

**[ASK ALL EVER USING (QO1b=1-2), SP]**

**QO4: Did you ever use [xxx] regularly, i.e. at least once per day?**

**PLEASE SELECT ONE ANSWER**

1. Yes
2. No

**[ASK IF EVER USED REGULARLY (Q02=1 OR QO4=1), NUMERIC, RANGE 1 – CURRENT AGE]**

**QO5: At what age did you start to use [xxx] regularly, i.e. at once per day?**

**PLEASE TYPE IN BELOW**

└─┴─┘years **[IF <= 15 SHOW POP UP MESSAGE: “Please confirm the age you have entered is correct and click next to continue" and allow to confirm this and proceed]**

Don’t know / can’t remember

**[ASK EX-USER (QO2=3), NUMERICS, YEAR RANGE 0 – CURRENT AGE, MONTH RANGE 0-11, DAY RANGE 0-30]]**

**QO6. For how long have you stopped your use of [xxx] now?**

**PLEASE PROVIDE AS MUCH INFORMATION AS POSSIBLE BY ENTERING THE YEARS, MONTHS AND DAYS AS APPROPRIATE**

└─┴─┘years plus └─┴─┘months plus └─┴─┘days

Don’t know / can’t remember

**[DISPLAY IF CURRENT OR OCCASIONAL USER EVER USING REGULARLY (QO2=1 OR (QO2=2 AND QO4=1))]**

**Please now think about any periods when you may have stopped regularly using [xxx] …**

**[DISPLAY IF EX-USER OR NON-USER THAT HAVE USED 100 TIMES OR MORE THAT REGULARLY USED ((QO2=3 OR (QO2=4 AND QO3=1)) AND QO4=1)]**

**Please now think about the time you first began using [xxx] to the present day …**

**[ASK IF EVER USED REGULARLY (Q02=1 OR QO4=1), NUMERICS, YEAR RANGE 0 – CURRENT AGE, MONTH RANGE 0-11, DAY RANGE 0-30]**

**QO7: [IF CURRENT USER EVER USING REGULARLY (QO2=1 OR (Q02=2 AND QO4=1))”If you ever stopped regular use of [xxx]: “]For how long did you stop altogether? Please add together any separate periods of stopping [IF EX-USER THAT REGULARLY USED (QO2=3 AND QO4=1)”, including the current period since you stopped your use of [xxx]”]**

**PLEASE PROVIDE AS MUCH INFORMATION AS POSSIBLE BY ENTERING THE YEARS, MONTHS AND DAYS AS APPROPRIATE [IF CURRENT OR OCCASIONAL USER EVER USING REGULARLY (QO2=1 OR (Q02=2 AND QO4=1)) “. IF YOU HAVE NEVER STOPPED PLEASE ENTER ZERO BELOW”]**

└─┴─┘years plus └─┴─┘months plus └─┴─┘days

Don’t know / can’t remember

**[DISPLAY IF EX-USER OR NON-USER THAT HAS USED 100 TIMES OR MORE (QO2=3 OR (QO2=4 AND QO3=1))]**

**Thinking now about the time when you used [xxx] …**

**[ASK ALL DAILY, OCCASIONAL OR EX-USER, OR NON-USERS THAT HAVE USED 100 TIMES OR MORE (QO2=1-3 OR (QO2=4 AND QO3=1)), SP]**

**QO8: What flavour of [xxx] did you predominantly use in the last 12 months of use?**

**PLEASE SELECT ONE ANSWER**

1. Regular / no flavour
2. Menthol
3. Other flavour
4. Don’t know / can’t remember

**[ASK ALL DAILY, OCCASIONAL OR EX-USER, OR NON-USERS THAT HAVE USED 100 TIMES OR MORE (QO2=1-3 OR (QO2=4 AND QO3=1)), SP]**

**QO9: On average, how many times do/did you use [xxx] per day?**

**Please indicate if you were on average USING IT NOT AT ALL, less than ONcE per day, or the number of TIMES per day at each of the time points below**

**[COLUMNS]**

1. Not at all
2. less than once per day
3. └─┴─┴─┘per day **[RANGE 1-200]**

**[ROWS, ONE ANSWER PER ROW]**

**[SUPRRESS ANY ROWS WHERE PARTICIPANT WOULD HAVE BEEN AGED 0 OR BELOW]**

1. Currently (last 3 months)
2. 1 year ago
3. 2 years ago
4. 5 years ago
5. 10 years ago
6. More than 10 years ago

**[ASK DAILY AND OCCASIONAL USERS (QO2=1-2), SP]**

**QO10: In total, how many times, if any, in the past 12 months have you tried to stop your use of [xxx] completely?**

**PLEASE SELECT ONE ANSWER**

1. I did not try to stop in the past 12 months
2. Please type in number of time(s)___ **[RANGE 1-365]**
3. Don’t know / can’t remember

**[ASK ALL WHO TRIED TO STOP USING COMPLETELY AT LEAST ONCE (QO10=2), NUMERICS, MONTH RANGE 0-11, DAY RANGE 0-30]**

**QO11: The last time you stopped your use of [xxx] in the past 12 months because you were trying to stop completely, how long did you stop in total?**

**PLEASE PROVIDE AS MUCH INFORMATION AS POSSIBLE BY ENTERING MONTHS AND DAYS AS APPROPRIATE**

└─┴─┘months plus └─┴─┘days

Don’t know / can’t remember

**[ASK CURRENT DAILY AND OCCASIONAL USERS (QO2=1-2), SP]**

**QO12: Are you considering stopping use of [xxx] during the next 6 months?**

**PLEASE SELECT ONE ANSWER**

1. Yes, plan to stop within next 30 days
2. Yes, plan to stop within next 6 months, but not within next 30 days
3. No, not thinking of stopping in next 6 months
4. Don’t know / couldn’t say

**[ASK DAILY AND OCCASIONAL USERS (QO2=1-2), MP 1-18, SP 19]**

**QO13: We are now interested in the reason(s) why you are using [xxx].**

**PLEASE REVIEW THE LIST OF POSSIBLE REASONS BELOW AND SELECT ALL THAT APPLY**

**I use [xxx]...**

**[RANDOMISE CODES 1-17]**

1. To quit tobacco use completely
2. To quit cigarette smoking
3. To reduce cigarette smoking without quitting completely
4. Because I believe it is less harmful than smoking cigarettes
5. Because I believe it is less addictive than smoking cigarettes
6. Because I believe it reduces the urge to smoke
7. Because I use it at locations where smoking cigarettes is prohibited
8. Out of curiosity
9. Because it is less expensive than smoking cigarettes
10. Because it is fun
11. Because of the various flavours available
12. Because friends, family or colleagues do so too
13. Because it tastes better to me than smoking cigarettes
14. Because it is cool/modern
15. Because I believe it bothers other people less than smoking cigarettes
16. Because it is hard for me to stop using [xxx]
17. Because people in the media or famous personalities use [xxx]
18. Other reasons
19. Don’t know / couldn’t say

**Other Tobacco or Nicotine Containing Products**

**[SECTION HEADING ABOVE NOT TO BE DISPLAYED TO PARTICIPANT]**

**The next few questions are about any experiences of using other products that contain tobacco or nicotine. (show products 4-6 images and text)**

**[ASK ALL, SP]**

**QP1: For each product listed below, please indicate your current use behaviour.**

**PLEASE SELECT ONE ANSWER FOR EACH PRODUCT BELOW**

**[COLUMNS]**

1. Daily use (using it at least once per day, disregarding religious fasting)
2. Occasional use (less than once per day)
3. Ex-user
4. Non-user

**[ROWS, ONE ANSWER PER ROW]**

1. Smokeless Tobacco (such as chewing tobacco, snus, snuff, dissolvable)
2. Other tobacco or nicotine-containing products (such as cigars, cigarillos, pipes, water-pipes)
3. Nicotine Replacement Therapy products also known as NRTs (such as, patches, gums, tablets, inhalers, lozenges, pills)

**First Products Used**

**[SECTION HEADING ABOVE NOT TO BE DISPLAYED TO PARTICIPANT]**

**QPROD (SYSTEM VARIBLE - NOT BE ASKED)**

**[DP – SYSTEM VARIABLE TO BE POPULATED AS FOLLOWS:]**

1. Cigarettes (including hand-rolled cigarettes) **[AUTOCODE IF QC1=1-3 OR QC2=1 OR QC3=1]**
2. IQOS with HEETS **[AUTOCODE IF QI1=1-3 OR QI2=1 OR QI3=1]**
3. Electronic cigarettes or vaping devices **[AUTOCODE IF (QO2=1-3 OR QO3=1 OR Q04=1) AND QO1b=1]**
4. Other innovative product **[AUTOCODE IF (QO2=1-3 OR QO3=1 OR Q04=1) AND QO1b=2]**
5. Smokeless Tobacco (such as chewing tobacco, snus, snuff, dissolvable) **[AUTOCODE IF QP1_1=1-3]**
6. Other tobacco or nicotine-containing products (such as cigars, cigarillos, pipes, water-pipes) **[AUTOCODE IF QP1_2=1-3]**
7. Nicotine Replacement Therapy products also known as NRTs (such as, patches, gums, tablets, inhalers, lozenges, pills) **[AUTOCODE IF QP1_3=1-3]**
8. None **[AUTOCODE IF QPROD = BLANK]**

**[ASK ALL EVER USING MORE THAN ONE NICOTINE PRODUCTS (QPROD = 1-7 AND MULTI-PUNCHED). IF QPROD=1-7 AND SINGLE PUNCHED, THEN AUTOCODE QF1 WITH PRODUCT CODED AT QPROD]**

**QF1: You mentioned that you are or were using the following product(s) below. Which ONE of these was the first product you ever used?**

**PLEASE SELECT ONE ANSWER**

1. Cigarettes (including hand-rolled cigarettes) **[DISPLAY IF QPROD=1]**
2. IQOS with HEETS **[DISPLAY IF QPROD=2]**
3. Electronic cigarettes or vaping devices **[DISPLAY IF QPROD=3]**
4. XXX **[INSERT VERBATIM ENTERED AT QO1b_2, DISPLAY IF QPROD=4]**
5. Smokeless Tobacco (such as chewing tobacco, snus, snuff, dissolvable) **[DISPLAY IF QPROD=5]**
6. Other tobacco or nicotine-containing products (such as cigars, cigarillos, pipes, water-pipes) **[DISPLAY IF QPROD=6]**
7. Nicotine Replacement Therapy products also known as NRTs (such as, patches, gums, tablets, inhalers, lozenges, pills) **[DISPLAY IF QPROD=7]**
8. Don’t know / can’t remember

**[ASK ALL IDENTIFYING PRODUCT USED FIRST (QF1=1-7), NUMERIC, RANGE 1 – AGE OF FIRST PRODUCT REGULARLY USED AT QF1)**

**QF2: At what age did you use xxx [INSERT PRODUCT CODED AT QF1] for the first time?**

**PLEASE TYPE IN BELOW**

└─┴─┘years

**Computed variable …**

**AGE_FIRST_USED [Computed variable – not be shown to participants]**

**IF QC4>=1 OR QI4>=1 OR Q05_1>=1 OR  Q05_2>=1, THEN RECODE AGE_FIRST_USED WITH THE MINIMUM POSITIVE VALUE GIVEN AT THESE QUESTIONS

[IF QF1=1,2,3,4,5,6 OR 7 AND AGE_FIRST_USED >= 1 AND QF2 >  AGE_FIRST_USED PLEASE SHOW THIS ERROR MESSAGE “Age has to be equal or lower than when you started using any product regularly”]**

**[IF QF1=1,2,3,4,5,6 OR 7 AND IF <= 15 SHOW POP UP MESSAGE: “Please confirm the age you have entered is correct and click next to continue" and allow to confirm this and proceed]**

Don’t know / can’t remember

**[ASK ALL IDENTIFYING PRODUCT USED FIRST (QF1=1-7), SP]**

**QF3: And still thinking about the xxx [INSERT PRODUCT CODED AT QF1] that you first used, what was the flavour this product?**

**PLEASE SELECT ONE ANSWER**

1. Regular / no flavour
2. Menthol
3. Other flavour
4. Don’t know / can’t remember

**General Health**

**[SECTION HEADING NOT TO BE DISPLAYED TO PARTICIPANT]**

**[DISPLAY FOLLOWING TEXT TO ALL PARTIPANTS]**

**The next few questions are about your general health …**

**[ASK ALL, SP]**

**QH1: How would you rate your overall health?**

**PLEASE SELECT ONE ANSWER**

1. Poor
2. Fair
3. Good
4. Excellent
5. Don’t know
6. Prefer not to say

**[ASK ALL, SP]**

**QH2: Compared to the average person your age, would you say your health is …?**

**PLEASE SELECT ONE ANSWER**

1. Much worse
2. Worse
3. The same
4. Better
5. Much better
6. Don’t know
7. Prefer not to say

**[ASK ALL, SP]**

**QH3: How much do you worry about your health?**

**PLEASE SELECT ONE ANSWER**

1. Not at all
2. Occasionally
3. Sometimes
4. Frequently
5. Don’t know
6. Prefer not to say

**[ASK ALL, MP 1-14, SP 15-16]**

**QH4a: The following is a list of common health problems. Which, if any, of these problems do you have?**

**PLEASE SELECT ALL THAT APPLY**

1. Heart disease
2. High blood pressure
3. Lung disease
4. Diabetes
5. Ulcer or stomach disease
6. Kidney disease
7. Liver disease
8. Anaemia or other blood disease
9. Cancer
10. Depression
11. Osteoarthritis, degenerative arthritis
12. Back pain
13. Rheumatoid arthritis
14. Other medical problems (please type in ….)
15. Do not have any medical problems
16. Prefer not to say

**[ASK ALL WITH MEDICAL PROBLEM (QH4a=1-14), MP 1-14, SP 15-16]**

**QH4b: And for which of these, if any, do you receive medication or some other type of treatment?**

**PLEASE SELECT ALL THAT APPLY**

**[DISPLAY MEDICAL PROBLEMS 1-14 CODED AT QH4a]**

1. Do not receive medication or treatment for any of the above
2. Prefer not to say

**[ASK ALL WITH MEDICAL PROBLEM (QH4a=1-14), M]**

**QH4c: And which, if any, limit any of your activities?**

**PLEASE SELECT ALL THAT APPLY**

**[DISPLAY MEDICAL PROBLEMS 1-14 CODED AT QH4a]**

1. None of the above limit my activities
2. Prefer not to say

**Socio-economic Questions (end of interview)**

**[SECTION HEADING NOT TO BE DISPLAYED TO PARTICIPANT]**

**[DISPLAY FOLLOWING TEXT TO ALL UK PARTIPANTS]**

**The last few questions are about you and your household.**

**[ASK ALL, SP]**

**UK_D1: What is the highest level of education that you have completed?**

**PLEASE SELECT ONE ANSWER**

1. GCSE/O-Level/CSE
2. Vocational qualifications (=NVQ1+2)
3. A-Level or equivalent (=NVQ3)
4. Bachelor Degree or equivalent (=NVQ4)
5. Masters/PhD or equivalent
6. Other
7. No formal qualifications
8. Still studying
9. Prefer not to say

**[ASK ALL, SP]**

**UK_D2: Which of the following best describes your working status?**

**PLEASE SELECT ONE ANSWER**

1. Have paid job - Full time (30+ hours per week)
2. Have paid job - Part time (8-29 hours per week)
3. Have paid job - Part time (Under 8 hours per week)
4. Not working – homemaker / looking after children
5. Self-employed (full time)
6. Self-employed (part time)
7. Full time student
8. Unemployed and seeking work
9. Retired
10. Not in paid work because of long term illness or disability
11. Not in paid work for other reason
12. Prefer not to say

**[ASK ALL WORKING OR SELF-EMPLOYED (D2=1,2,3,5,6), SP]**

**UK_D3: Which of the following best describes your occupation or profession?**

**PLEASE SELECT ONE ANSWER**

1. Professional (doctor, lawyer, architect, etc.)
2. Senior Management / Executive
3. Senior Civil Servant
4. Middle Management
5. Local Government / Civil Service Officer
6. Education (teacher, lecturer, etc.)
7. Services/Military
8. Supervisor / Junior management
9. Owner of small establishment
10. Clerical/office worker (including customer services etc.)
11. Skilled manual (plumbers, electricians, etc.)
12. Semi-skilled manual (including security work, driver, carer, etc.)
13. Un-skilled manual (including retail, sales assistant etc.)
14. Apprentice
15. Other, write in: __________________________
16. Prefer not to say

**[ASK ALL, SP]**

**UK_D4: Which of these categories best describes your total combined household income for the past 12 months?**

**PLEASE SELECT ONE ANSWER**

1. Up to £9,499
2. £9,500 - £17,499
3. £17,500 - £29,999
4. £30,000 - £49,999
5. £50,000 or more
6. Prefer not to say

**[ASK ALL, NUMERIC 1-30]**

**UK_D5A: How many people, in all, live in your household, including yourself? Please exclude any domestic staff, paying guests or family members away for over 6 months**

**PLEASE TYPE IN NUMBER OF PERSON(S)**

___

Prefer not to say

**[ASK ALL LIVING IN MULTI-PERSON HOUSEHOLD (UK_D5=2-30), NUMERIC 0 - UK_D5A- 1]**

**UK_D5B: And how many of those, if any, are under 18 years of age?**

**PLEASE TYPE IN NUMBER OF PERSON(S)**

___person(s)

Prefer not to say

**[ASK ALL, SP]**

**UK_D6: Do you have British citizenship?**

**PLEASE SELECT ONE ANSWER**

1 Yes

2 No

3 Prefer not to say

**[ASK ALL, SP]**

**UK_D7: What is your ethnicity?**

**PLEASE SELECT ONE ANSWER**

- 1. White
  2. Black Caribbean
  3. Black African
  4. Other Black groups
  5. Indian
  6. Pakistani
  7. Bangladeshi
  8. Chinese
  9. Another Ethnic group
  10. Prefer not to say

**[DISPLAY FOLLOWING TEXT TO ALL PARTIPANTS]**

**Thank you for taking the time to complete our survey. Please return the laptop to the interviewer.**

**[TO BE ASKED BY INTERVIEWER AT THE END OF THE INTERVIEW]**

**[ASK ALL, SP]**

**UK_D8: Social Grade Module**

- Occupation Of The Chief Income Earner In Household (Present Or Last)
- Job Title (Present Or Last)
- Qualifications / Apprenticeships (Enter Verbatim)
- Number Responsible For (Enter Number)

(Enter Verbatim - Allow DK or Refused)

**[TO BE COMPLETED BY THE INTERVIEWER: CODE SOCIAL GRADE A, B, C1, C2, D, E]**

**[PLACE HOLDER FOR CLOSING SCRIPT]**
