# Supplementary material for: Household Surveys in the General Population and Web-Based Surveys in IQOS Users Registered at the Philip Morris International IQOS User Database: Protocols on the Use of Tobacco- and Nicotine-Containing Products in Germany, Italy, and the United Kingdom (Greater London), 2018-2020
Source: JMIR Res Protoc. 2019 May 9;8(5):e12061. doi: 10.2196/12061 (PMC6532333; doi:10.2196/12061)
Supplement: Multimedia Appendix 2 [file resprot_v8i5e12061_app2.docx]

**Appendix 2 Additional Questionnaires for IQOS Users Survey in Greater London**

**Screening questions**

**[ASK ALL, SP]**

**S4: Are you currently using *IQOS* with *HEETS*?**

**PLEASE SELECT ONE ANSWER**

1. Yes
2. No

**[close & thank if No]**

**[ASK ALL, SP]**

**S5: Have you ever used 100 *IQOS* *HEETS or more* in your life?**

**PLEASE SELECT ONE ANSWER**

1. Yes
2. No

**[close & thank if No]**

**[ASK ALL, SP]**

**S6: Do you currently work for Philip Morris International or any of its affiliates?**

**PLEASE SELECT ONE ANSWER**

1. Yes
2. No

**[close & thank if YES]**

**[CLOSE & THANK MESSAGE]**

**“Thank you for your interest in this study. However, based on the information provided, you do not qualify for participation. Thank you for your cooperation up to this point”**

**Misuse Questions**

**[DISPLAY FOLLOWING TEXT]**

**The next few questions are about how you use IQOS with HEETS.**

**[ASK ALL, SP]**

**Q1a: Have you ever consumed an IQOS HEETS without using the IQOS device?**

**PLEASE SELECT ONE ANSWER**

1. Yes
2. No

**[ASK ALL CONSUMING HEETS WITH IQOS DEVICE (Q1a=2), MP]**

**Q1b: When consuming an IQOS HEETS without the IQOS device, what did you do?**

**PLEASE SELECT ALL THAT APPLY**

1. I lit up the HEETS like a cigarette
2. I used the HEETS with another device
3. I used it in some other way

**[ASK ALL LIGHTING UP THE HEETS LIKE A CIGARETTE (Q1b=1), SP]**

**Q1c: How often have you lit up HEETS like a cigarette?**

**PLEASE SELECT ONE ANSWER**

1. Only once
2. Sometimes
3. Most of the time
4. All the time

**[ASK ALL USING HEETS WITH ANOTHER DEVICE (Q1b=2), SP]**

**Q1d: How often have you used HEETS with another device?**

**PLEASE SELECT ONE ANSWER**

1. Only once
2. Sometimes
3. Most of the time
4. All the time

**[ASK ALL USING HEETS IN SOME OTHER WAY (Q1b=3), SP]**

**Q1e: How often have you used HEETS “in some other way”?**

**PLEASE SELECT ONE ANSWER**

1. Only once
2. Sometimes
3. Most of the time
4. All the time

**[ASK ALL, SP]**

**Q2: How often, if at all, have you used the IQOS device with a product other than HEETS?**

**PLEASE SELECT ONE ANSWER**

1. Never
2. Only once
3. Sometimes
4. Most of the time
5. All the time

**Risk Perception Questions**

**[DISPLAY FOLLOWING TEXT]**

**This section asks for your views about the risks of using IQOS. After reading each question, select the answer that best reflects how you feel, keeping in mind that there are no right or wrong answers. Based on the information you have read about IQOS, please answer all questions. If you are unsure about how to answer a question, give the best answer that you can.**

**[ASK ALL]**

**Q3: In general, what do you think would be the risk, if any, to users of IQOS with HEETS of getting the following (sometime during their lifetime) because of using IQOS?**

**PLEASE ANSWER FOR EACH BELOW**

**[COLUMNS]**

1. No risk
2. Low risk
3. Moderate risk
4. High risk
5. Very high risk
6. Don’t know

**[ROWS, ONE ANSWER PER ROW, DISPLAY OF 3 SCREENS WITH 6 STATEMENTS TO APPEAR ON EACH SCREEN]**

1. having a bad cough that lasts for days
2. having poor gum health
3. having lung cancer
4. occasional wheezing (difficult breathing that produces a sound)
5. having mouth or throat cancer
6. ageing faster (for example, wrinkles on the face)
7. being sick with frequent minor illnesses (for example, coughs and colds)
8. having regular respiratory infections (for example, bronchitis, pneumonia)
9. having a serious illness (for example, chest pain, vascular disorder, diabetes)
10. having reduced stamina
11. having emphysema (serious lung disease)
12. having a cough early in the morning
13. losing some sense of taste
14. having heart disease
15. an earlier death
16. having sores of the mouth or throat
17. being physically unfit
18. having other types of cancer (besides mouth, throat, or lung)

**[DISPLAY FOLLOWING TEXT]**

**This survey asks for your views about the risks of smoking cigarettes. After reading each question, select the answer that best reflects how you feel, keeping in mind that there are no right or wrong answers. Please answer all questions. If you are unsure about how to answer a question, give the best answer that you can.**

**[ASK ALL]**

**Q4: In general, what do you think is the risk, if any, to smokers of getting the following (sometime during their lifetime) because of smoking cigarettes?**

**PLEASE ANSWER FOR EACH BELOW**

**[COLUMNS]**

1. No risk
2. Low risk
3. Moderate risk
4. High risk
5. Very high risk
6. Don’t know

**[ROWS, SP PER ROW, DISPLAY OF 3 SCREENS WITH 6 STATEMENTS TO APPEAR ON EACH SCREEN]**

1. having a bad cough that lasts for days
2. having poor gum health
3. having lung cancer
4. occasional wheezing (difficult breathing that produces a sound)
5. having mouth or throat cancer
6. ageing faster (for example, wrinkles on the face)
7. being sick with frequent minor illnesses (for example, coughs and colds)
8. having regular respiratory infections (for example, bronchitis, pneumonia)
9. having a serious illness (for example, chest pain, vascular disorder, diabetes)
10. having reduced stamina
11. having emphysema (serious lung disease)
12. having a cough early in the morning
13. losing some sense of taste
14. having heart disease
15. an earlier death
16. having sores of the mouth or throat
17. being physically unfit
18. having other types of cancer (besides mouth, throat, or lung)

**Self-Reported Changes Questionnaire (SRCQ)**

**[ASK ALL EX-SMOKER OF COMBUSTIBLE CIGARETTES (QC1=3)]**

**Q5: Thinking of your experience since you switched to IQOS with HEETS, please indicate to what extent you agree or disagree with the following statements.**

**PLEASE ANSWER FOR EACH BELOW**

**[COLUMNS]**

1. Strongly Disagree
2. Disagree
3. Somewhat disagree
4. Do not agree or disagree
5. Somewhat agree
6. Agree
7. Strongly Agree
8. Don’t know / couldn’t say

**[ROWS, ONE ANSWER PER ROW]**

Compared to when I was smoking combustible cigarettes …

1. The skin on my face appears smoother and firmer
2. I feel that it is easier to exercise (e.g., run and walk)
3. My sense of smell has improved
4. My sense of taste has improved
5. I feel/my partner feels, my breath smells better
6. My teeth appear less stained or yellowish

**Tobacco/Nicotine-containing Product Evaluation Questionnaire**

**[ASK ALL, SP]**

**Q6a: Have you used IQOS with HEETS today?**

**PLEASE SELECT ONE ANSWER**

1. Yes
2. No

**[ASK ALL USING IQOS TODAY (Q6a=1)]**

**IQ6b: After reading each question, please select the answer that best reflects how using IQOS HEETS made you feel today, keeping in mind that there are no right or wrong answers. Please answer all questions. If you are unsure about how to answer a question, give the best answer that you can.**

**PLEASE ANSWER FOR EACH BELOW**

**[COLUMNS]**

1. Not at all
2. Very little
3. Little
4. Moderately
5. A lot
6. Quite a lot
7. Extremely

**[ROWS, ONE ANSWER PER ROW]**

1. Was it satisfying?
2. Did it taste good?
3. Did you enjoy the sensations in your throat and chest?
4. Did it calm you down?
5. Did it make you feel more awake?
6. Did it make you feel less irritable?
7. Did it help you concentrate?
8. Did it reduce your hunger for food?
9. Did it make you dizzy?
10. Did it make you nauseated?
11. Did it immediately relieve your craving for IQOS?
12. Did you enjoy it?
